# Bayesian dose selection design for a binary outcome using restricted response adaptive randomization

**DOI:** 10.1186/s13063-017-2004-6

**Published:** 2017-09-08

**Authors:** Caitlyn Meinzer, Renee Martin, Jose I. Suarez

**Affiliations:** 1 Data Coordination Unit, Department of Public Health Sciences, Medical University of South Carolina, 135 Cannon Street, Charleston, SC USA; 2 Division of Neurocritical Care, Departments of Anesthesiology and Critical Care Medicine, Neurology, and Neurosurgery, Johns Hopkins University, Houston, TX USA

**Keywords:** Dose selection, Response adaptive randomization, Phase II, Adaptive design, Bayesian design, Clinical trial

## Abstract

**Background:**

In phase II trials, the most efficacious dose is usually not known. Moreover, given limited resources, it is difficult to robustly identify a dose while also testing for a signal of efficacy that would support a phase III trial. Recent designs have sought to be more efficient by exploring multiple doses through the use of adaptive strategies. However, the added flexibility may potentially increase the risk of making incorrect assumptions and reduce the total amount of information available across the dose range as a function of imbalanced sample size.

**Methods:**

To balance these challenges, a novel placebo-controlled design is presented in which a restricted Bayesian response adaptive randomization (RAR) is used to allocate a majority of subjects to the optimal dose of active drug, defined as the dose with the lowest probability of poor outcome. However, the allocation between subjects who receive active drug or placebo is held constant to retain the maximum possible power for a hypothesis test of overall efficacy comparing the optimal dose to placebo. The design properties and optimization of the design are presented in the context of a phase II trial for subarachnoid hemorrhage.

**Results:**

For a fixed total sample size, a trade-off exists between the ability to select the optimal dose and the probability of rejecting the null hypothesis. This relationship is modified by the allocation ratio between active and control subjects, the choice of RAR algorithm, and the number of subjects allocated to an initial fixed allocation period. While a responsive RAR algorithm improves the ability to select the correct dose, there is an increased risk of assigning more subjects to a worse arm as a function of ephemeral trends in the data. A subarachnoid treatment trial is used to illustrate how this design can be customized for specific objectives and available data.

**Conclusions:**

Bayesian adaptive designs are a flexible approach to addressing multiple questions surrounding the optimal dose for treatment efficacy within the context of limited resources. While the design is general enough to apply to many situations, future work is needed to address interim analyses and the incorporation of models for dose response.

## Background

In the context of a drug development program, the goals of a phase II clinical trial are arguably the most nebulous and most critical. In phase I, the number of subjects exposed to a drug are limited, and the goals are most often focused on assessing safety and identifying a maximum tolerated dose. As the drug progresses to phase III, the number of resources (cost, sites, subjects) is significantly increased to definitively test whether the new drug is superior to a control using a clinically meaningful outcome. A chasm exists between these phases in which investigators must resolve numerous scientific and practical questions, including whether the maximum tolerated dose is also the maximum effective dose, and whether the selected dose shows sufficient promise to justify expending the resources for a phase III trial.

The definition of “dose” can have multiple components — intensity, duration, frequency, and whether the drug is administered as a constant volume or some variable algorithm (e.g., loading and maintenance doses). Furthermore, the choice of dose may be defined as discrete options or on a continuous range. It is impossible to investigate all of these aspects in a single trial. Investigators must decide, based on the scientific premise and previously collected safety data, which dose should be evaluated for a signal of efficacy. Several approaches have been suggested in the literature, including phase I/II trials that search over a range of doses to identify the dose which optimizes safety and efficacy [1], methods that rank and select from a set of candidate doses [2], and hybrid designs that incorporate both aspects [3]. Regardless of the specific design, a trade-off exists between the breadth and depth of information collected about the efficacy of the doses under consideration.

Although several authors [4–6] have shown that testing more treatments with fewer subjects per arm increases the probability of identifying an efficacious treatment, this also leads to less precision in the resulting estimate of treatment effect. Response adaptive randomization (RAR) designs have been proposed as a more ethical alternative by which patients are more likely to be randomized to the best performing arm [7, 8]. In the context of dose selection, RAR also allows investigators to collect more information on the dose(s) that are most likely to proceed forward to phase III, though at the cost of loss of information in other regions of the dose-response curve [9, 10]. There is not, however, a consensus that RAR is an improvement over fixed allocation when comparing a single treatment to control; critics note that the adaptive algorithm results in reduced power to test a hypothesis comparing the selected treatment arm to the control arm [11]. As a result, several authors have proposed constrained RAR algorithms that allow more subjects to be allocated to the best performing arm while retaining desirable study design characteristics such as power [12–15]. Finally, note that it is assumed here that a trial using RAR is based on an endpoint which is directly relevant to patient health. (For a robust discussion on the ethics of RAR, see [16]).

The current manuscript considers a trial wherein investigators wish to first select from a range of possible active doses before evaluating whether the selected dose is sufficiently promising when compared to a control group to warrant a phase III trial. Borrowing from the adaptive design literature, the proposed design uses RAR to allocate subjects to the most promising dose. However, throughout the duration of the study, the number of subjects allocated to the active doses versus control is held constant to protect the power for a test comparing the selected active dose arm and the control arm. The proposed design is described in detail below. To provide context for the design, an example trial for subarachnoid hemorrhage is described. Subsequently, different parameterizations of the design are considered and their relative properties discussed.

### Study design

**Fig. 1:**
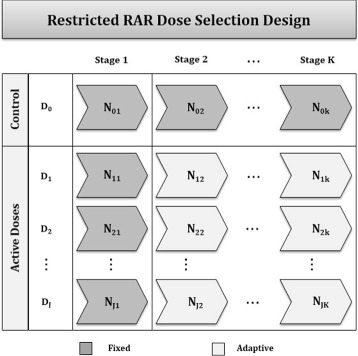
Generalized Bayesian response adaptive allocation design for dose selection, where the allocation between control and active doses is held constant

Consider a trial where investigators wish to randomize a total of *N* subjects to a control arm (*D*_0_) and *J* active dose arms (*D*_1_,…,*D*_*J*_) at each of *K* stages, with the goal of assessing whether the active treatment can reduce the proportion of bad outcomes in the disease of interest. In the proposed design (Fig. 1), the total number of subjects is partitioned in the following ways, where *N*_*jk*_ represent the number of new subjects randomized to the *j* ^*th*^ arm at the *k* ^*th*^ stage.

First, the proportion of subjects randomized to the control arm and the pooled active arms is held at a constant ratio throughout the trial. That is,

$$ \frac{N_{01}}{{\sum\nolimits}_{j=0}^{J} N_{j1}} = \ldots = \frac{N_{0K}}{{\sum\nolimits}_{j=0}^{J} N_{jK}} = \frac{{\sum\nolimits}_{k=1}^{K}N_{0k}}{{\sum\nolimits}_{k=1}^{K}{\sum\nolimits}_{j=0}^{J} N_{jk}}, $$

and

$$ \frac{{\sum\nolimits}_{j=1}^{J} N_{j1}}{{\sum\nolimits}_{j=0}^{J} N_{j1}} = \ldots = \frac{{\sum\nolimits}_{j=1}^{J} N_{jK}}{{\sum\nolimits}_{j=0}^{J} N_{jK}} = \frac{{\sum\nolimits}_{k=1}^{K} {\sum\nolimits}_{j=1}^{J} N_{jk}}{{\sum\nolimits}_{k=1}^{K}{\sum\nolimits}_{j=0}^{J} N_{jk}}. $$

Second, to ensure that estimates of the effect size for each active dose are stable, a burn-in period is required wherein the number of subjects allocated to each of the active doses is fixed during stage 1. This number is prespecified to reflect the desired trade-off between the risk of randomizing subjects to a suboptimal dose and the degree of flexibility in the design. Thus, for stage 1, *N*_11_=…=*N*_*J*1_. For stages 2 through *K* the number of subjects randomized to each of the active doses is:

$$ N_{jk} = r_{jk}N_{.k}, \;\;\;0 \leq r_{jk} \leq 1,\;\; \sum\limits_{j=1}^{J} r_{jk} =1 $$

where *r*_*jk*_ is the allocation ratio to the *j* ^*th*^ active dose at the *k* ^*th*^ stage as defined by the RAR allocation ratio, and *N*_.*k*_ is the total number of subjects allocated to the active dose arms for stage k, that is, $N_{.k}={\sum \nolimits }_{j=1}^{J}N_{jk}$.

The following sections describe how to estimate the treatment effect for each arm and set the RAR algorithm, as well as how the choice of partition for active and control arms, fixed and adaptive stages, and aggressiveness of the RAR algorithm will affect the design properties. For simplicity, it is assumed that each of the adaptive stages are equally spaced, *N*_02_=…=*N*_0*K*_ and ${\sum \nolimits }_{j=1}^{N} N_{j2} = \ldots = {\sum \nolimits }_{j=1}^{N} N_{jK}$. It is straightforward to generalize the design to the case of interim updates that are not equally spaced.

## Methods

For Bayesian trials, the fundamental unit of analysis is the posterior probability of an event for each of the arms to which a subject may be randomized, *D*_0_,…,*D*_*J*_. From this, secondary quantities can be derived, including the probability that each active dose yields the maximal effect, the probability that a treatment difference comparing an active dose to the control is greater than a minimal clinically significant difference, and the relative allocation of subjects. The approach to calculating these quantities is first discussed, followed by a comparison of their application in different RAR schemes.

### Posterior probability of treatment effect

The primary outcome measure is the proportion of subjects who experience an event within the follow-up period, where the goal of treatment is to reduce this proportion when compared to the proportion of events in the control arm. As is true of any trial, the treatment effect in each arm can be estimated using simple summary statistics (i.e., the proportion of events in each arm) or as estimates from a model, with dose modeled alternatively as a categorical or continuous function. Here, the simple case is presented where the effect of each dose is estimated independently. Generalizations to model-based methods of estimation are discussed in the “More complex dose selection algorithms” section.

The prior probability of observing an event in each of the *j* treatment arms has an independent uniform beta distribution:

$$ \pi\left(\theta_{j}\right)\sim Beta(1,1), j=0,\ldots,J. $$

This distribution is chosen to be uninformative, and can be alternatively interpreted as having observed two subjects worth of data where one subject has an event and the other does not. At the *k* ^*th*^ analysis, *Y*_*j*_ total subjects have experienced the event of interest out of *N*_*j*_ total subjects randomized to the *j* ^*th*^ arm, which can be described as a binomial likelihood where *Y*_*j*_ and *N*_*j*_ are taken to represent the sum of events up to the *k* ^*th*^ stage (i.e., *N*_*j*_=*N*_*j*1_+…+*N*_*jk*_, and *Y*_*j*_=*Y*_*j*1_+…+*Y*_*jk*_). Thus, the posterior probability distribution of an event in each arm is

$$ \pi\left(\theta_{j}|Y_{j},N_{j} \right)\sim Beta\left(1+Y_{j},1+N_{j}-Y_{j}\right). $$

Given the posterior probability for each treatment arm, it is now possible to calculate the probability that each active dose is the most effective (i.e., the basis of the RAR algorithm) and the probability that a given dose, when compared to control, is sufficiently promising for further investigation (i.e., the primary outcome).

### Response adaptive randomization (RAR) algorithm

The goal of the RAR algorithm is to randomize the maximum number of subjects to the most effective active dose, *D*_1_,…,*D*_*J*_. Initial estimates of the posterior probability are made using the data observed from the *N*_*j*1_ subjects enrolled to each of the *j*>0 active doses in stage 1. For each subsequent stage, *k*=2,…,*K*, the RAR algorithm updates the allocation ratios *r*_*jk*_ such that more subjects are randomized to the dose which has the greatest probability of a favorable outcome (i.e., a smaller proportion of events).

#### Probability of maximum treatment effect

After each of the *k* stages, the probability that each active dose yields the maximum treatment effect (i.e., the greatest reduction in poor outcomes), denoted *P*(*θ*_*j*_=*θ*_*min*_), is estimated as:

$$\begin{array}{@{}rcl@{}} \begin{aligned} P\left(\theta_{1}=\theta_{min}\right) & = \int_{0}^{1} \pi\left(\theta_{1}|Y_{1},N_{1}\right)P\left(\theta_{2},\ldots,\theta_{j}>\theta_{1}\right)\\&\quad \quad \partial \theta_{J}\ldots \partial\theta_{2} \partial\theta_{1}\\ &\,=\, \int_{0}^{1}\!{\pi \left(\theta_{1}|Y_{1},N_{1}\right)}\! \int_{\theta_{1}}^{1}{\pi\left(\theta_{2}|Y_{2},N_{2}\right)} \cdots \! \int_{\theta_{1}}^{1}{\pi\left(\theta_{J}|Y_{J},N_{J}\right)}\\&\quad \quad \partial \theta_{J}\ldots \partial\theta_{2} \partial\theta_{1} \end{aligned} \end{array} $$

for the case of *θ*_1_, with *θ*_2_.…*θ*_*j*_ being specified similarly:

$$\begin{array}{@{}rcl@{}} \begin{aligned} P\left(\theta_{2}\,=\,\theta_{min}\right) & \,=\, \int_{0}^{1}\!{\pi \left(\theta_{2}|Y_{2},N_{2}\right)} \int_{\theta_{2}}^{1}\!{\pi\left(\theta_{1}|Y_{1},N_{1}\right)} \cdots \int_{\theta_{2}}^{1}{\pi\left(\theta_{J}|Y_{J},N_{J}\right)}\\&\quad \quad \partial \theta_{J}\ldots \partial\theta_{1} \partial\theta_{2} \\ &\vdots& \\ P\left(\theta_{J}=\theta_{min}\right) & =\int_{0}^{1}{\pi \left(\theta_{J}|Y_{J},N_{J}\right)} \int_{\theta_{J}}^{1}{\pi\left(\theta_{1}|Y_{1},N_{1}\right)} \cdots \int_{\theta_{J}}^{1}\\&\quad{\pi\left(\theta_{(J-1)}|Y_{(J-1)},N_{(J-1)}\right)} \quad \partial \theta_{(J-1)}\ldots \partial\theta_{1} \partial\theta_{J}. \\ \end{aligned} \end{array} $$

The probability is estimated through simulation from the empirical distribution. Note that the interest is in estimating which active dose is the most effective; as a result, it is not necessary to compare the active doses to the control group in this calculation.

#### RAR algorithm

Although the goal of the RAR algorithm is to allocate the majority of subjects to the most promising active dose arm, if the algorithm is overly sensitive to spurious trends in the observed outcome rates, there is an increased risk of allocating an excess of subjects to a suboptimal dose. At stage *k* we define a raw weight *I*_*jk*_ for each active dose arm, *j*>0, as a function of the probability that the arm is the best, and the standard error of the treatment effect, where the sensitivity of the algorithm is determined by two tuning parameters, *γ* and *λ*:

$$I_{jk}=\left[P\left(\theta_{j}=\theta_{min}\right)\right]^{\gamma} \left[\frac{Var\left(\theta_{j}\right)}{\left(N_{j}+1 \right)}\right]^{\lambda}. $$

Note that this equation is comparable to other RAR algorithms currently proposed in the literature [17–19].

Once the raw weight *I*_*jk*_ has been estimated for each active dose, the allocation ratio is rescaled so the final ratio *r*_*jk*_ for each active dose arm sums to 1. That is, for stage *k*:

$$ r_{jk}=\frac{I_{j}}{{\sum\nolimits}_{j>1}^{J} I_{j}} $$

When *γ*,*λ*=0 the RAR allocation ratios are equivalent to fixed equal allocation among the doses (i.e., *I*_*j*_=1 which implies *r*_*jk*_=1/(*J*−1)).

### Primary analysis

The primary analysis is based on the posterior probability that the absolute risk difference of an event, comparing the selected dose to the control, is greater than a minimum clinically interesting difference, *C*. Here, the selected dose is identified as the dose most likely to reduce the number of poor outcomes. That is, define the primary analysis as *P*(*θ*_*j*_−*θ*_0_>*C*), where *j*= max[*P*(*θ*_*j*_=*θ*_min_)], and the posterior probability is calculated as:

$${{} \begin{aligned} P\left(\theta_{j}-\theta_{0} >C\right) &= \int_{0}^{1} \pi\left(\theta_{1}|Y_{1},N_{1}\right)\\ &\quad\times \int_{0}^{\theta_{j}-C} \pi\left(\theta_{0}|Y_{0},N_{0}\right) \quad \partial \theta_{0}\partial\theta_{j}. \end{aligned}} $$

If this posterior probability exceeds the prespecified cutoff, *ε*, the null hypothesis *H*_0_:*θ*_*j*_−*θ*_0_>*C* is rejected with *ε*100% level of certainty. For instance, *P*(*θ*_*j*_−*θ*_0_>0)>0.8 can be interpreted as at least 80% probability that the absolute risk difference is greater than 0 (i.e., 80% probability that the selected dose is no worse than the control).

Finally, note here that while this procedure makes no explicit assumptions about the relative ranking of the effect size for each active dose, *D*_1_,…,*D*_*j*_, it is implied that the dose *j* which satisfies *j*= max[*P*(*θ*_*j*_=*θ*_min_)] also maximizes *P*(*θ*_*j*_−*θ*_0_>*C*). This is true so long as *θ*_0_≤{*θ*_1_,…,*θ*_*j*_}. Similarly, because the posterior probability is one-sided, this is true regardless of whether the effect size in the control is better than some doses and worse than others (e.g., *θ*_1_<<*θ*_0_<*θ*_2_<…<*θ*_*j*_). However, if the posterior probability were structured as a two-sided hypothesis (e.g., *P*(*θ*_*j*_−*θ*_0_≠*C*)), it would still be desirable to select *j*= max[*P*(*θ*_*j*_=*θ*_min_)] despite no longer maximizing the power to reject the null hypothesis.

## ALISAH II: albumin in subarachnoid stroke

The impetus for this design is a phase II trial in subarachnoid hemorrhage, a common neurological emergency with high morbidity and mortality rates, and several common neurological complications including aneurysm rebleeding, hydrocephalus, and delayed cerebral ischemia [20]. Despite the number of patients affected by subarachnoid hemorrhage and the poor prognosis, few treatments have been developed to improve outcomes and reduce hospital stay [21]. Recent clinical trials investigating compounds such as endothelin-1 antagonists and magnesium sulfate have led to disappointing negative results [22–24]. The failure of these clinical trials may be due in part to the fact that most of the treatments tested are posited to have limited mechanisms of action. Given the complex cerebral cascade of events unleashed by subarachnoid hemorrhage, the administration of a multifunctional substance with the potential to target multiple pathways is compelling. Intravenous administration of 25% human albumin (referred to hereafter as albumin) is one such potential treatment.

The Albumin in Subarachnoid Hemorrhage Pilot Clinical Trial (ALISAH, ClinicalTrials.gov registration number NCT01747408) was a prospective, open-label, dose escalation study examining four dosages of albumin in increasing magnitude [25]. In this study, albumin was administered for up to 7 days, and an optimal dose of 1.25 g/kg/d of albumin was identified as safe, feasible to administer, and associated with reduced resource utilization and possible neuroprotective effects [25, 26]. However, evidence from both the ALISAH pilot trial [25] and the Albumin in Acute Stroke trial (ALIAS, Parts 1 and 2, ClinicalTrials.gov registration number NCT00235495) [27–29], which evaluated albumin in ischemic stroke patients, suggested that administration of albumin could have deleterious cardiovascular effects due to volume overload [25, 29]. Thus, although the ALISAH pilot study demonstrated that the selected dose administered for up to 7 days was tolerable, questions remain regarding the optimal efficacious duration of continuous infusion. Specifically, it is possible that a longer duration of infusion may be associated with increased adverse events (e.g., acute left heart failure due to volume overload) [25–29].

In the currently proposed study (ALISAH II), investigators want to identify the optimal duration (1, 3, 5, or 7 days) of albumin to administer in subjects with subarachnoid hemorrhage and test whether there is preliminary evidence of efficacy for this optimal duration when compared to a saline infusion control. The goal of treatment is to reduce the proportion of subjects with poor outcome, defined as an Extended Glasgow Outcome Scale score ≤4 observed at 90 days. The proposed phase II Bayesian restricted RAR design is presented in the context of the ALISAH II trial development with specific focus on the key design considerations.

## Results

To calculate the total sample size, several quantities must be specified a priori including the expected rate of events in the control group, the clinically meaningful effect difference, and the desired design properties (i.e., type I error rate, power, and/or probability of correctly selecting the best dose) [30]. For simple designs, this information is sufficient. However, for the proposed design, there is no closed-form equation for the total sample size, nor is it immediately obvious how to allocate the total sample size among the arms or stages given a total sample size. Instead, an iterative approach is used to first identify a crude estimate for the total sample size, which is then optimized using a series of diagnostic measures.

### Total sample size

For phase II trials, a Go, No-Go criteria is predefined as the minimum level of evidence to warrant a phase III trial. That is, for this design the study results would support progressing to phase III with the selected dose if *P*(*θ*_*min*_−*θ*_0_>*C*) exceeds a prespecified level. Thus, a key operating characteristic is the probability that the trial will achieve this threshold under null and alternative distributions for treatment effect (i.e., the type I error rate and power).

**Fig. 2:**
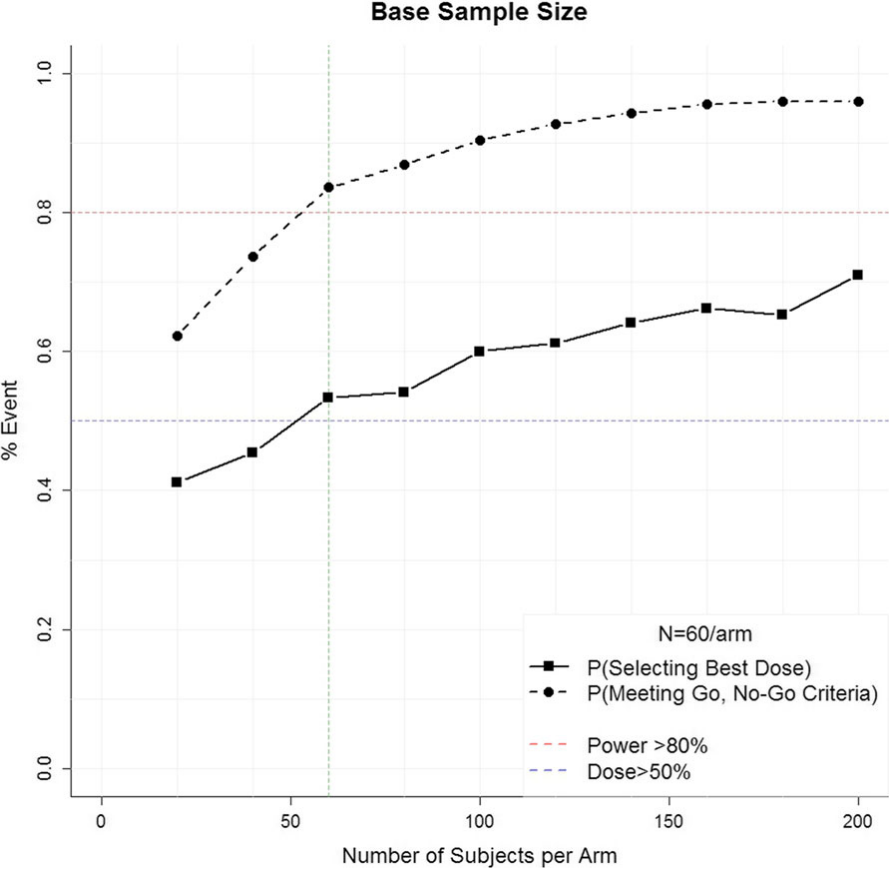
Crude estimation of the total sample size needed to achieve the minimum probability of selecting the optimal dose and meeting the Go, No-Go criteria

A crude estimate of the total sample size is first estimated by fixing the number of stages as *K* = 1 and equally allocating the total number of subjects among the four active arms and control (i.e., *N*_01_=*N*_11_=…=*N*_*J*1_=*N*/5). The total sample size is the minimum *N* that yields the desired probability of selecting the best dose and probability of meeting the Go, No-Go criteria under the alternative hypothesis. Figure 2 demonstrates this process for ALISAH II, where investigators hypothesize that the event rate in the control group will be 28%, the clinically meaningful reduction is 10%, the effect rate among the doses is linear (i.e., 25.5%, 23%, 20.5%, 18%), and there is a minimum 50% probability of selecting the correct dose (blue line) and an 80% probability of meeting the Go criteria (*P*(*θ*_*j*_−*θ*_0_>0)>0.8, red line). With 1000 simulations each, sample sizes ranging from 20 subjects per arm to 200 subjects per arm were considered.

A total of 60 subjects per arm was identified as the minimum sample size that achieves the desired properties. Note that as the sample size per arm increases, the probability of meeting the Go, No-Go criteria (i.e., power) increases until approximately 60 subjects, after which point there are diminishing returns of adding additional subjects. However, if selecting the optimal dose is of higher priority, it would be reasonable to select a larger sample size regardless of the relatively minimal additional gain in power. Finally, note that for the adaptive design to be worthwhile, it must perform better than this simple approach.

### Design optimization: allocation ratio, number of stages, and burn-in

Within the constraints of total sample size (*N* = 60/arm) and the basic proposed design (Fig. 1), additional simulations were conducted to assess the global design properties: ability to select the optimal dose and probability of proceeding to phase III under different specifications. A range of possible design choices for the number of stages, allocation between control and pooled active doses, and choice of tuning parameters for RAR were assessed, with each scenario repeated for 1000 iterations to obtain estimates.

**Fig. 3:**
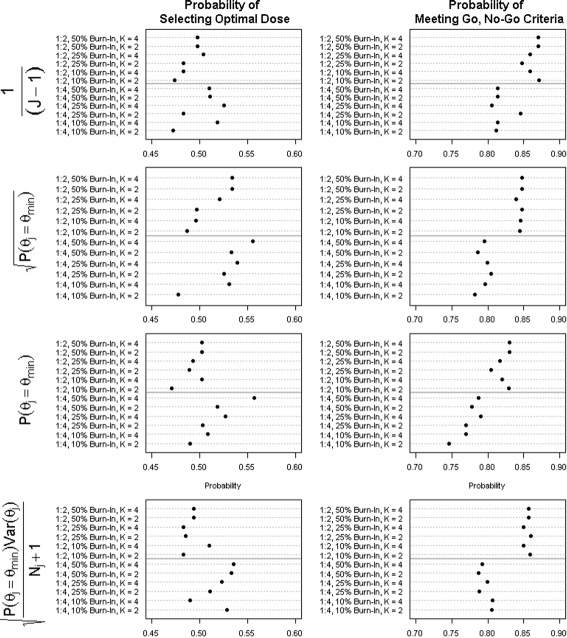
Under the alternative hypothesis of a linear treatment effect, the figure evaluates the effect of allocation between treatment and control, number of stages, and percentage of active subjects used in stage I on the global design properties: the probability of passing the Go, No-Go criteria and the probability of correct dose selection. Results are presented for four possible crude weights, *I*_*jk*_ of the RAR specifications

Figure 3 demonstrates how different allocations of subjects among the stages and arms affect the probability of selecting the optimal dose (here 7 days, assuming a linear dose response) and the probability that the primary analysis will reject the null hypothesis (i.e., pass the Go, No-Go criteria and proceed to phase III). Note that a 1:4 allocation ratio would be equivalent to equally allocating among the five arms if no RAR is used; similarly, 1:2 would be equivalent to a $1:\sqrt {J}$ allocation ratio (i.e., Dunnett’s allocation [31]). As the total number of subjects allocated to the active doses increases from 1:2 to 1:4, the probability of selecting the correct dose increases. However, the probability of meeting the Go, No-Go criteria decreases. For a given allocation ratio, the percentage of active subjects allocated to the fixed burn-in period (stage 1) is positively correlated with the probability of selecting the optimal dose. However, there is not a strong correlation between the percentage of burn-in subjects and the probability of meeting the Go, No-Go criteria. Finally, the optimal number of RAR updates (i.e., increased number of stages) is strongly dependent on the other design choices, with a slight tendency towards increased updates yielding better properties. Given these results, a 25% burn-in and *k*=4 stages offered the best balance of dose selection and power.

### Design optimization: RAR algorithm

**Fig. 4:**
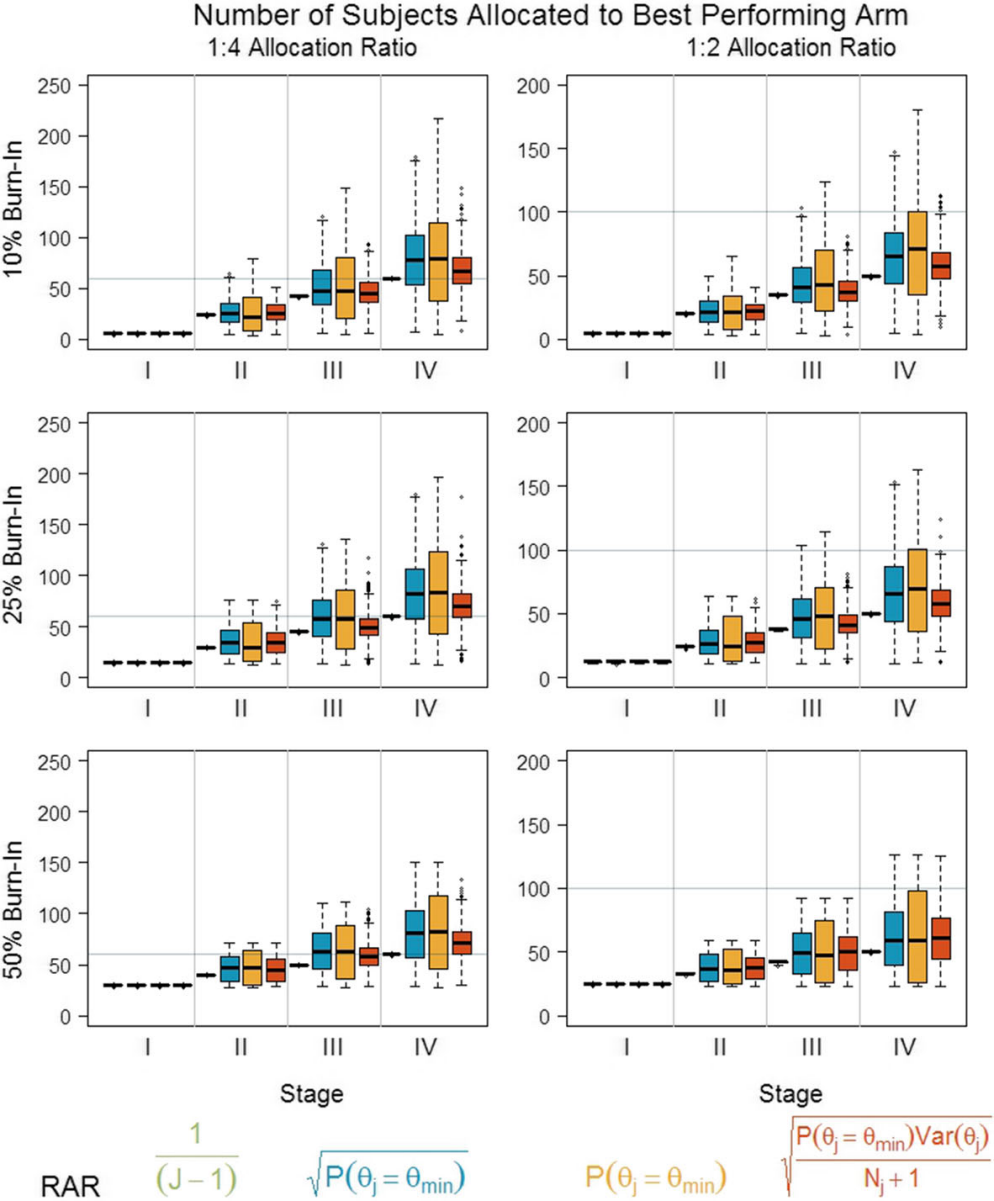
Number of subjects allocated to the best performing arm as a function of the percentage of subjects allocated to the burn-in stage, the allocation ratio, and the RAR algorithm. *Horizontal gray line* represents the number of subjects allocated to the control group and thus the target number of subjects allocated to the optimal dose to achieve a 1:1 allocation for the primary hypothesis test

In addition to identifying the optimal allocation ratio of subjects between control and active doses, fixed and adaptive stages, and the number of adaptive updates, the choice of the RAR algorithm has a significant impact on the design properties. Figures 3 and 4 display the properties for four possible raw RAR weights, *I*_*jk*_: No RAR (*γ*=0,*λ*=0), using only the probability that an active dose yields the minimum treatment effect with tuning parameter of either 1/2 or 1 (*γ*=1/2 or *γ*=1,*λ*=0), and a raw weight that also incorporates the variability of the estimate with a tuning parameter of 1/2 (*γ*=1/2,*λ*=1/2). When no RAR is used, the choice of number of stages or burn-in period is trivial because the number of subjects in each arm is equal under all possible specifications. For all other scenarios, the design is sensitive to the choice of RAR; however, no general statements can be made (Fig. 3). Moreover, while the results presented here consider only the case of a linear trend, the optimal RAR algorithm will be dependent on the hypothesized shape and spread of the dose-response distribution.

The literature notes that while frequent updates (i.e., obtained by increasing the number of stages) will increase the responsiveness to true treatment effects, there is an increased risk that subjects may be allocated to the worse performing arm due to an ephemeral treatment effect [17]. Thus, in addition to the global design properties specified above, the number of subjects assigned to each treatment arm at each stage is assessed (Fig. 4). In each plot, the horizontal line indicates the total number of subjects allocated to the control group. An optimal RAR algorithm would have a mean total number of subjects in the highest dose that yields a final ratio of 1:1, with the smallest possible variance. For the hypothesized trend, fixed allocation (i.e., no RAR, 1/(*J*−1)) minimizes the variability in the number of subjects allocated to the best arm (and thus reduces the number of subjects allocated to suboptimal doses). However, because it does not incorporate accumulating information on the dose-response trend, it also consistently allocates the fewest number of subjects to the optimal arm relative to the other algorithms. The two algorithms which randomize subjects solely based on the treatment effect (*γ*=1/2 or *γ*=1,*λ*=0) allocate more subjects to the best dose compared to no RAR. However, particularly in the scenario with a *γ*=1 tuning parameter, the high variability suggests that it is still possible to allocate subjects to a suboptimal arm (though rarely less than if no RAR were used). The algorithm that incorporates the estimate variability (*γ*=1/2,*λ*=1/2) performs slightly better than no RAR; the separation between doses is insufficient to overcome the estimate of variability, and thus the optimal dose does not receive the majority of subjects. Finally, note that although there are differences in the number of subjects allocated to the optimal dose between algorithms, there are no significant differences in the probability of meeting the Go, No-Go criteria for all of the RAR algorithms except the algorithm with tuning parameters *γ*=1,*λ*=0 (Fig. 3). That is, unlike the two-arm scenario (but similar to other multi-arm RAR designs [32]), we do not experience a loss in power by allocating more subjects to the optimal active arm. Thus, for the linear dose trend, the algorithm that only accounts for the treatment effect but modulates the strength through a *γ*=1/2 tuning parameter represents the best balance between optimal design parameters and an overly sensitive RAR algorithm.

## Discussion

The motivation for the ALISAH II trial was discussed, and a design with 25% burn-in, four stages, and an RAR algorithm with tuning parameters of *γ*=1/2,*λ*=1/2 was selected to optimize the trial properties if the probability of poor outcome in the albumin doses was linear. The choice of an optimal design is dependent on the hypothesized magnitude and relationship of effect between the doses. A linear dose-response trend is the most difficult in terms of dose selection; thus, a design that increases the number of observations used to estimate the Go, No-Go criteria helps mitigate a smaller effect size if a suboptimal but still effective dose is selected. The above simulations would need to be repeated to identify the optimal design if a different dose-response relationship were hypothesized, though the general themes are consistent.

The following section now considers whether the design is sufficiently optimal for additional dose-response relationships and discusses further possible modifications the study team might consider. Table 1 presents four possible outcomes in the trial: (a) when all four active doses are ineffective (i.e., 28% poor outcome rate in the control, day 1, day 3, day 5, day 7); (b) when only the maximum dose duration is effective (i.e., 28% poor outcome rate in the control, day 1, day 3, and day 5, but 18% poor outcome rate in day 7); (c) when there is a linear dose-response trend ranging from 28% in the control arm to 18% in the 7 day arm; and (d) when all four active doses are equally effective (i.e., 18% poor outcome rate). For each of these scenarios, the following statistical properties are presented: the observed treatment effect; the number of subjects allocated to the optimal dose, defined alternately as the dose with the greatest effect size or the shortest duration in the case of two or more equivalent doses (i.e., 1 day for scenarios a, d; 7 days for scenarios b, c); the probability of selecting the correct dose; the unconditional power, defined as the number of scenarios where the posterior probability of a treatment effect exceeds 80% regardless of the dose selected; and finally, the conditional power defined similarly to the unconditional power but restricted to those iterations where the correct dose was selected. Each scenario was simulated for 10,000 iterations under the assumption of 100 subjects in the control arm and 200 subjects among the active arms.

**Table 1:** Operating characteristics for different treatment effect assumptions: (a) all four active doses are ineffective; (b) only the maximum dose duration is effective; (c) linear dose-response trend; and (d) all four active doses are equally effective

	Tx effect	*N*	*P*(Dose)	Uncond. power	Cond. power
a	29.0	48	24.9	41.0	41.2
b	18.4	85	78.7	76.1	81.6
c	18.8	64	50.3	84.3	87.5
d	19.1	47	24.2	93.8	94.0

The table presents the median observed effect size for the optimal dose arm (Tx effect), the average number of subjects randomized to that arm (*N*) and the unconditional and conditional power defined respectively as the percentage of simulations where *P*(*θ*_*j*_−*θ*_0_>0)≥0.8 conditional and unconditional on whether *j* was the true optimal dose

When there is no difference between the four active dose arms (scenarios a, d), the median number of subjects allocated is equivalent to the scenario where no RAR is used (i.e., the 200 subjects are randomized equally among the four doses for approximately 50 subjects per arm). Similarly, the probability of selecting the correct arm is about 25%. When the doses are not equally effective, the use of RAR outperforms the no RAR scenario with the optimal dose (7 days) receiving more subjects and a higher probability of selecting the correct dose than chance alone. Note that, as expected, when only one dose is effective, the task of identifying the optimal dose is easier, as reflected in the greater number of subjects and probability of picking that dose. However, in scenario c, while the probability of selecting the best dose is almost 20% less than if a single optimal dose exists, the probability of selecting the best or second best dose is much higher (results not shown). This is desirable, as the cost of selecting the second best dose is much less than if a dose with no efficacy were selected.

To decide whether the current evidence is sufficiently promising to continue with a future phase III trial, the Go, No-Go criteria are defined as at least 80% probability that the probability of poor outcome in the selected active dose is less than the probability of poor outcome in the control arm (i.e., *P*(*θ*_*j*_−*θ*_0_<0)≥80*%*). This is conceptually similar to the frequentist concept of type I error if the treatments are simulated under the null (scenario a), or power if under the alternative (scenarios b–d). Secondly, this quantity can be evaluated conditional or unconditional on having selected the correct dose, where the former is equivalent to the assumptions made for non-adaptive trials (i.e., separate trials for dose finding and testing of efficacy), and the latter is more typical of adaptive frameworks. Note that, by selecting for the optimal dose, the design introduces a positive bias in the treatment effect for the selected active dose [33, 34]. Although this is a well-known problem in adaptive selection designs, it is not straightforward to predict the magnitude of the bias or adjust the resulting statistics. Nevertheless, as long as the trial properties are transparent, the inflated power is still a useful quantity for decision making. Here, when at least one dose is effective, there is high conditional power to proceed to phase III. Unconditional power is similarly high but reflects the risk of selecting a suboptimal dose. In the null case, the probability of proceeding to phase III when no treatment effect exists, 40%, is higher than would normally be accepted for a phase II trial. Historically, the use of type I error levels has been accepted as an immutable requirement, although more recently discussion in the statistical community has acknowledged that the type I error rate should reflect the trade-offs specific to a trial, and as such may be higher in certain situations [6, 35, 36]. Given the severity of the patient population and the lack of available treatment options, there is a greater harm of stopping after the phase II trial given an effective treatment than to proceed to future testing with an ineffective treatment (particularly given the ability to use futility boundaries in the phase III trial). In this light, approximately 60% of futile phase III trials would be prevented, but only 5–20% of effective phase III trials would be lost. However, if in other disease contexts this trade-off is unacceptable, improved properties can be achieved by increasing the total sample size, increasing the minimum acceptable difference, or making the threshold to proceed to phase III more strict.

In the context of designing the ALISAH II trial, the most basic aspects of implementing the restricted RAR design have been presented. One of the strengths of this approach, however, is its flexibility. Although it is beyond the scope of the current paper to evaluate in detail how additional modifications would affect the probability of selecting an optimal dose which also meets the Go, No-Go criteria, what follows is a discussion of the main possibilities and their expected effect.

### More complex dose selection algorithms

It may be desirable to consider a more complex dose selection rule wherein, for instance, the shortest duration which achieves at least 90% of the maximum effect size is selected (i.e., an ED-90 type rule). Clinically, this would be the case if the higher dose significantly decreases the feasibility of implementation or is associated with increased risk of harm. To implement this rule, the dose selection criteria of select *j*=*max*[*P*(*θ*_*j*_=*θ*_*min*_)] could be replaced with *j*=*min*(*j*)∈*P*(*θ*_*j*_≥0.9(*θ*_*min*_)), though additional simulations would be required to identify the optimal RAR algorithm in this scenario (e.g., randomize equally to all doses meeting the ED-90 duration or proportional to a “relative-value” weight). It is anticipated that this selection rule would perform similarly to the simple rule in the case of a clear winner, but have improved ability to select the optimal dose when there are small differences in the effect size. Conversely, since a less than maximally efficacious dose may be promoted, the power may be reduced.

The model presented in the “Methods” section assumes the observed probability of poor outcomes has a simple binomial distribution; however, in practice there are often covariates that modulate this effect. For instance, in ALISAH II it is anticipated that baseline stroke severity will be a significant predictor of outcome. Here, a hierarchical logistic regression model could replace the current binomial distribution. The resulting posterior probability of poor outcome in each arm could then be compared using the above RAR algorithms and Go, No-Go criteria, with likely mild to moderate improvement in the design properties. It is important to note, however, that optimal methods for simultaneous covariate adaptive and response adaptive randomization are relatively new and, generally speaking, a tight balance of covariates will diminish the benefit of RAR.

Once a hierarchical logistic regression model has been considered, another logical extension is the inclusion of effects in the model which no longer treat the doses as categorical but rather introduce polynomial terms (e.g., a linear dose-response trend) and/or borrow information between the doses (e.g., similar to a normodynamic linear model). As above, this is relatively simple to implement, though it requires careful consideration of the appropriate priors for each coefficient in the model. However, the use of a dose-response model will significantly increase the number of simulation scenarios that are required. That is, in addition to the considerations above, it will also be necessary to evaluate the model’s performance if the dose-response shape is incorrectly specified. In practice this is well worthwhile, as it is likely that the resulting improvement in dose selection and power is non-trivial.

## Conclusions

The proposed Bayesian adaptive phase II design is a flexible approach to balancing the need to identify the optimal dose from several possible doses and making a statement with a certain level of confidence about the relative efficacy when compared to a control group. Several design components were introduced (allocation of subjects, choice of burn-in, RAR algorithm), and simulation results were presented showing how these aspects can affect the resulting statistical design properties. An example design was considered for the case of albumin use for patients with subarachnoid hemorrhage. However, the design is broadly applicable for trials where the primary endpoint is binary and two or more active treatments are available (i.e., the treatments may be doses or even different drugs). One limitation of this design is that it requires the primary endpoint to be available in a timely manner relative to the rate of recruitment, or an appropriate validated surrogate endpoint should exist. If not, too many subjects are recruited before a sufficient number of outcomes are observed to implement the RAR.

While the design does not include any additional features such as interim analyses for efficacy and futility, or logistic regression models that leverage the additional information available from the non-selected arms or measurements of the primary outcome at earlier time points, the authors posit that these additions are straight-forward, and in the case of modeling will improve the overall design properties so long as the choice of model is reasonable. Moreover, the statistical design properties presented above are conservative, and use of these additional features will likely improve the final interpretation of results.

## Abbreviations

ALISAH: Albumin in subarachnoid hemorrhage
RAR: Response adaptive randomization
